# Balloon venoplasty opens the road for an implantable defibrillator patient with complex stenosis

**DOI:** 10.1002/ccr3.1002

**Published:** 2017-05-16

**Authors:** Peter Magnusson, Robert Kastberg

**Affiliations:** ^1^ Cardiology Research UnitDepartment of MedicineKarolinska InstitutetStockholmSE‐171 76Sweden; ^2^ Centre for Research and DevelopmentUppsala University/Region GävleborgGävleSE‐ 801 87Sweden

**Keywords:** Balloon, implantable cardioverter–defibrillator, pacemaker lead, venoplasty, venous occlusion, venous stenosis

## Abstract

There is an increasing need for physicians to handle venous obstructions in pacemaker/implantable cardioverter‐defibrillator implants. Venoplasty performed by an experienced operator is a simple, safe, and fast way to manage this situation and proceed to implant. Compared to other approaches, this strategy may offer particular advantages.

## Introduction

Globally, more than one million pacemakers and 200,000 implantable cardioverter‐defibrillators (ICDs) are implanted every year 1. Accordingly, the number of complications will increase and patients with venous obstruction will become a clinical burden over time 2. With the increasing need for reinterventions, including the placement of new leads following lead failure or device upgrade, a strategy for the management of venous obstruction is warranted. This may involve skills and consultations that are outside the routine of the device laboratory. We report, after informed consent, a case of venoplasty, which allowed for the implantation of an ICD‐DR system despite multiple complex obstructions of the vein.

## Case History

A 77‐year‐old man underwent a complete extraction of an ICD device system due to endocarditis and was referred for a new ICD system. The patient had a primary prevention indication for ICD owing to a prior myocardial infarction and subsequent ischemic cardiomyopathy with a decreased left ventricular ejection fraction (EF).

His EF was estimated on echocardiography at 30% and confirmed with scintigraphy (24%), which showed evidence of irreversible ischemia. Percutaneous coronary intervention (PCI) had first been performed 16 years ago and another procedure 6 years ago left the patient with three stents. In addition to ischemic heart disease, the patient also had hypertension, diabetes mellitus, and renal insufficiency (glomerular infiltration rate 37 mL/h). He was pharmacologically managed with metoprolol, ramipril, aldactone, acetylsalicylic acid, insulin, and atorvastatin. There was no history of atrial fibrillation and the ECG showed QRS width 96 msec. Two active‐fixation leads, a 65 cm single‐coil Durata™, and a 58 cm Optisense™ lead (St. Jude Medical, St. Paul, MN) were implanted in the right ventricular apex and the right atrial appendage, respectively. Follow‐up at the device clinic showed normal sensing, impedance, and thresholds. Over the course of 5 years, the amount of atrial pacing increased (98%) but <1% ventricular pacing was observed.

**Figure 1:**
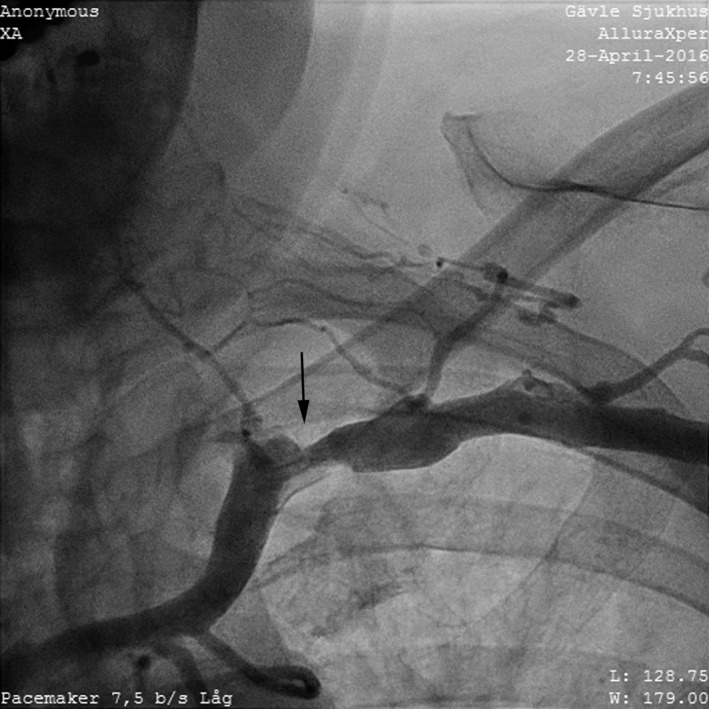
Venogram of left subclavian distal stenosis.

**Figure 2:**
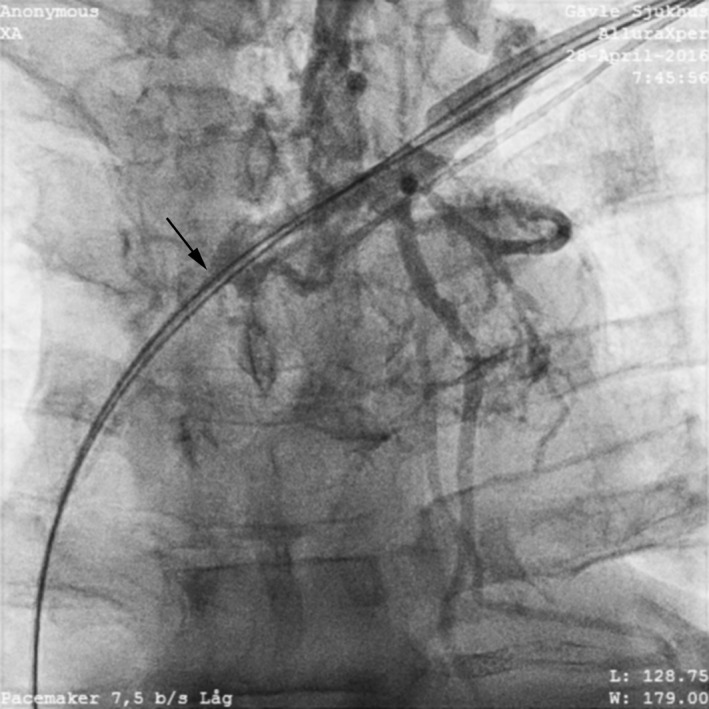
Venogram of left subclavian proximal stenosis.

**Figure 3:**
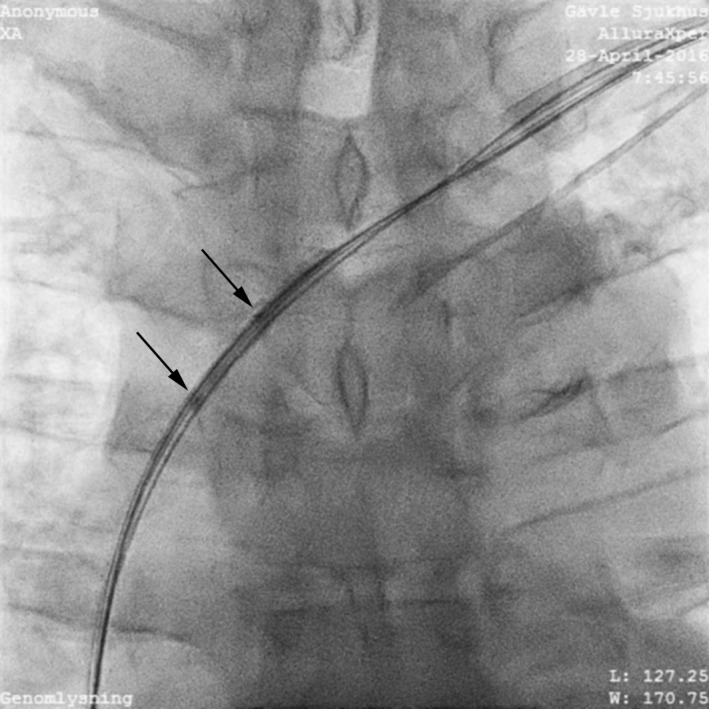
Balloon dilatation of left subclavian proximal stenosis.

**Figure 4:**
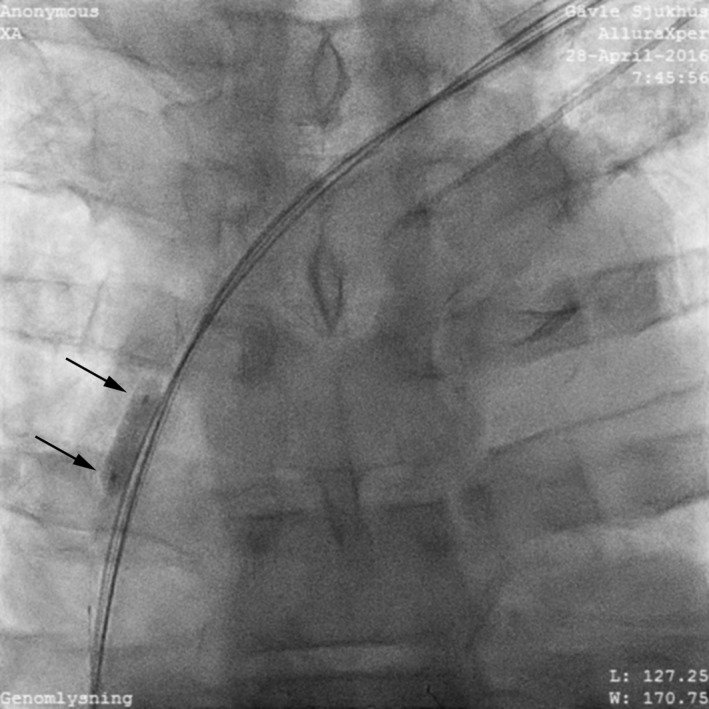
Balloon dilatation of left subclavian proximal stenosis.

Unfortunately, the patient suffered a *Staphylococcus aureus* infection with fever and a transoesophageal echocardiogram revealed suspect vegetation of the leads. The device system was completely explanted. Following persistent elevated inflammatory markers and prolonged antibiotic regimen, the implantation of a new ICD system was scheduled (Figs 1, 2, 3, 4).

A venogram was performed at the beginning of the procedure, which showed a partial occlusion of the proximal portion of the left subclavian vein and a tight lesion in the mid‐portion of superior vena cava. The cephalic vein was sacrificed at the first implant, so venous access was achieved from the axillary vein. This was successful on first attempt and using a 9.5 French introducer set with a retained guidewire, a single‐coil Optisure™ 58 cm lead (St Jude Medical) was easily affixed in the right ventricular apex. However, even with an eight French introducer it was impossible to pass through the subclavian vein to the superior vena cava. For that reason, 5 mL contrast was flushed into the introducer and a venogram revealed an occluded vein. A separate axillary vein puncture was performed, and an eight French introducer was inserted but could not be passed through the occluded area. We attempted to pass a thinner, six French lead, but only after several attempts using a Sion Blue™ guidewire (Asahi, Kardia Medical, Inc., Winnipeg, ON, Canada) was passage achieved.

An over the wire balloon Monorail 1.50 × 30 mm (Boston Scientific, Natick, MA) was inserted and inflated to 16 atm and then to 3.00 × 30 mm. Because the lesion was deemed short, a Maverick™ 5.0 × 15 mm (Boston Scientific) was subsequently used to dilate the narrowest portions of the lesion. The duration of the balloon venoplasty was 12 min.

Following venoplasty, the second lead could be easily passed down into the right atrium, and a 52 cm Tendril™ (St Jude Medical) was affixed in the atrial appendage wall. Sensing, impedance, and threshold values were satisfactory, and both leads were then connected to the Ellipse™ DR device (St. Jude Medical). The patient was discharged the next morning, and connected to a home‐monitoring system. His follow‐up in the clinic at 4 weeks was completely normal.

## Discussion

We report on a case in which cooperation between device and PCI operators using the equipment typically reserved for coronary interventions allowed us to complete a successful balloon venoplasty and subsequently implant an ICD‐DR in a patient with significant venous stenosis. In up to half of pacemaker implantations, significant occlusions due to fibrosis and/or thrombus will be encountered 3, and severe occlusion is found in 11% of cases 4. Mechanical wall stress causes inflammation and fibrosis, which can ultimately result in venous obstruction. In ICD patients, total venous occlusion is found in 9% of patients; up to 25% of patients show some degree of stenosis 4.

Occlusion of the superior vena cava encountered during device implantation is rarely treated, but the literature reports cases of successful surgery and stenting 5. We routinely perform a venogram before puncture in patients with a history of vessel procedures, including device upgrades or re‐implant after device extraction. These venograms guide our decision regarding optimal venous access. Blind puncture should be avoided, as there is an increased risk of accidental puncture of an artery or a nontarget vein, which may result in hematoma that can be difficult to differentiate from occlusion.

In this case, the first lead (defibrillation lead) was passed successfully, and we did not expect to have any difficulties with the second atrial lead. This patient required an atrial lead as he had sick sinus syndrome and was on beta‐blockade and thus needed atrial pacing support to achieve atrioventricular (AV) synchrony.

In our hospital, the coronary intervention laboratory and device surgery laboratory are next door to each other. This makes collaboration easy, and this balloon venoplasty in our case was performed by an experienced operator who has performed more than 3,000 coronary interventions.

There are a number of approaches that may be useful to overcome the challenges presented by occluded veins.

### Single‐lead system

In some cases, it may be helpful to reconsider the indication for a dual‐chamber system. In the absence of a bradycardia indication, an ICD‐VR may be an option. However, even then an atrial lead may be necessary to help discriminate from a supraventricular tachycardia. The physician must also take into account that even if the patient may be able to rely on a single‐lead system today, a revision to a dual‐chamber system may be necessary in the future–and this device surgery will be much more complicated.

### Contralateral implant of a secondary lead

This approach requires tunneling the lead over the often‐thin subcutaneous tissue at the sternum. Tunneling of the lead in this manner can be quite difficult and will delay the implant procedure substantially. From a long‐term perspective, tunneling a lead will complicate future implants (bilateral occlusion), a factor that is especially important to consider in patients with a long‐life expectancy 6.

### Right‐sided implantation

While this approach overcomes the disadvantages of tunneling, it brings with it the same periprocedural difficulties. If a right‐sided approach is used, it is highly recommended to exclude occlusion or venous anomalies from the right side before attempting venous access. The physician must also recognize that a pneumothorax from both sides may have devastating consequences. While pacemakers have been routinely implanted from the right side, right‐sided ICD implants are much rarer. For one thing, ICDs are larger and bulkier which can complicate a right‐sided implant, and more importantly, right‐sided ICD implantation can alter the shocking vector in such a way that increases the defibrillation threshold 7, 8, 9.

### Recanalization with/without extraction using sheaths and/or laser techniques

This approach requires specialized equipment and very specific skills. The use of these techniques may be safer in a center with thoracic surgical backup including available extracorporeal perfusion. The extraction sheath could damage existing leads, and in the case of infection all leads are routinely removed 8, 10.

### Medial puncture

This method may be considered as an alternative approach, but puncture near or of the superior vena cava may result in serious complications, such as pneumothorax or puncture of the trachea or a proximal artery 11.

### Open‐chest surgery

This approach requires a thoracic surgeon. Historically, epicardial leads have been placed successfully in open‐chest approaches. Short‐term and long‐term complications of this approach are postsurgical pain and a higher rate of lead failures, respectively.

### Subcutaneous ICD implantation

These novel devices may be used as an alternative system in those situations where transvenous access is impossible. While a subcutaneous ICD can be implanted and offers defibrillation capabilities, it lacks certain features. For example, a subcutaneous ICD cannot deliver antitachycardia pacing to terminate episodes of ventricular tachycardia. A subcutaneous ICD cannot provide pacing support, so it will not be a viable option for patients with symptomatic bradycardia. There are only limited data available for the long‐term use of such devices 12, 13. The subcutaneous device is bulkier than a conventional ICD, but future down‐sizing may alleviate this problem. Discrimination algorithms in subcutaneous devices are not as sophisticated as those in transvenous systems. Thus, device features must be carefully considered if a subcutaneous ICD is chosen.

### Alternative routes

Pacemaker leads, more often than ICD leads, have been successfully implanted using alternative routes which may be appropriate in certain selected ICD patients. The jugular vein route has been used historically, but results in an increased risk of complications due to tunneling over the collar bone; this is only an option if the obstruction does not significantly obstruct the vena cava lumen. Femoral venous access and a femoral/abdominal pocket can be used, but this requires longer leads and may increase the risk of instability. Furthermore, pocket discomfort and erosion may also occur in an abdominal implant. An innovative new approach called “inside‐out access” using femoral venous access combined with anterior venipuncture of the occluded subclavian vein has been described. However, complications due to central venous access might occur 14.

### A leadless pacemaker

These new devices can be inserted from the femoral vein for right ventricular pacing or via the femoral artery for left ventricular pacing 15. The experience with leadless pacemakers has been limited, so there are no long‐term data in larger series available. Furthermore, the cost of these new devices is also considerably higher than the costs of conventional systems 15.

Considering the different options, we believe that balloon venoplasty should be considered in the case of venous occlusion. If performed by an experienced operator, this approach appears to be both fast and convenient 16, 17, 18. Safety aspects should be very carefully considered, and sound clinical judgment is required. Many potential vascular obstructions can be handled using stents or temporary balloon inflation, while seeking assistance from a vascular/thoracic surgeon. It is advisable to consider different possible options beforehand, especially when the patient presents with a high probability for venous occlusion, such as patients who need device upgrades or revisions after endocarditis. The cooperation of a pacemaker operator and a coronary angiography operator may result in successful lead implant, even when severe or complex obstruction is encountered.

## Conclusions

Balloon venoplasty is a safe and simple approach to overcome venous obstruction during pacemaker/ICD lead implantation. It may be advocated as an alternative instead of right‐sided implants, tunneling, jugular or femoral access, or scheduling epicardial lead surgery. When considering an implant where venoplasty might be required, it is beneficial to work with an experienced angioplasty operator and to assure that the necessary technical resources and devices are available.

## Authorship

PM: involved in writing of the article and clinical management of the patient. RK: involved in the venoplasty and revision of the article. Both authors approved the final version of the case report for submission to the *Clinical Case Reports*.

## Conflict of Interest

None declared.
